# MAP Kinase Phosphatase-2 Plays a Critical Role in Response to Infection by *Leishmania mexicana*

**DOI:** 10.1371/journal.ppat.1001192

**Published:** 2010-11-11

**Authors:** Mashael S. Al-Mutairi, Laurence C. Cadalbert, H. Adrienne McGachy, Muhannad Shweash, Juliane Schroeder, Magdalena Kurnik, Callum M. Sloss, Clare E. Bryant, James Alexander, Robin Plevin

**Affiliations:** 1 Division of Physiology & Pharmacology, Strathclyde Institute of Pharmacy & Biomedical Sciences, University of Strathclyde, Glasgow, United Kingdom; 2 Division of Infection, Immunity and Microbiology, Strathclyde Institute of Pharmacy & Biomedical Sciences, University of Strathclyde, Glasgow, United Kingdom; 3 Department of Veterinary Medicine, University of Cambridge, Cambridge, United Kingdom; Imperial College London, United Kingdom

## Abstract

In this study we generated a novel dual specific phosphatase 4 (DUSP4) deletion mouse using a targeted deletion strategy in order to examine the role of MAP kinase phosphatase-2 (MKP-2) in immune responses. Lipopolysaccharide (LPS) induced a rapid, time and concentration-dependent increase in MKP-2 protein expression in bone marrow-derived macrophages from MKP-2^+/+^ but not from MKP-2^−/−^ mice. LPS-induced JNK and p38 MAP kinase phosphorylation was significantly increased and prolonged in MKP-2^−/−^ macrophages whilst ERK phosphorylation was unaffected. MKP-2 deletion also potentiated LPS-stimulated induction of the inflammatory cytokines, IL-6, IL-12p40, TNF-α, and also COX-2 derived PGE_2_ production. However surprisingly, in MKP-2^−/−^ macrophages, there was a marked reduction in LPS or IFNγ-induced iNOS and nitric oxide release and enhanced basal expression of arginase-1, suggesting that MKP-2 may have an additional regulatory function significant in pathogen-mediated immunity. Indeed, following infection with the intracellular parasite *Leishmania mexicana*, MKP-2^−/−^ mice displayed increased lesion size and parasite burden, and a significantly modified Th1/Th2 bias compared with wild-type counterparts. However, there was no intrinsic defect in MKP-2^−/−^ T cell function as measured by anti-CD3 induced IFN-γ production. Rather, MKP-2^−/−^ bone marrow-derived macrophages were found to be inherently more susceptible to infection with *Leishmania mexicana*, an effect reversed following treatment with the arginase inhibitor nor-NOHA. These findings show for the first time a role for MKP-2 *in vivo* and demonstrate that MKP-2 may be essential in orchestrating protection against intracellular infection at the level of the macrophage.

## Author Summary

In cells of the immune system are switch-on enzymes called kinases which regulate responses to infectious agents such as *Leishmania*. However, in the same cells there are switch-off enzymes known as phosphatases which function to turn off the kinases once they have done their work. A lot of studies have focussed on the role of kinases but not phosphatases in response to infection; we therefore generated a novel mouse in which the gene for one of these phosphatases, called MKP-2, has been deleted. We found that in the absence of this phosphatase unexpected things happened. The profile of inflammatory proteins, produced by a special cell of the immune system, called a macrophage, that functions to regulate infection by *Leishmania*, changed in ways which meant the macrophage could either fight infection very effectively or very weakly. In actual fact, we found that the macrophages with no MKP-2 fought off *Leishmania* poorly and mice deficient in MKP-2 had a modified immune response favouring the growth of the parasite. This is the first study to give critical insight into the role of MKP-2 in fighting *Leishmania* infection and demonstrates very well the importance of this class of enzyme in pathogen biology.

## Introduction

The mitogen-activated protein (MAP) kinase phosphatases (MKPs) are a family of dual specific phosphatases which regulate the functional activity of the major MAP kinase subfamilies through tyrosine and threonine dephosphorylation [1]. At least eleven isoforms exist each with different structures, subcellular distributions, substrate specificity and mechanisms of regulation. For example, the prototypic MKP-1 is induced by a wide variety of extracellular signals, is strictly nuclear located, and is able to dephosphorylate all MAP kinases, whereas MKP-3 is constitutively expressed, cytosolic and selective for extracellular regulated kinase (ERK) above the other major MAP kinases, c-Jun N-terminal kinase (JNK) and p38 MAP kinase. Thus, the action of one or more MKP is essential for the tight regulation of MAP kinase activity and subsequent functional responses mediated by a vast array of extracellular stimuli [1].

A number of MKPs have been implicated in the regulation of disease. Dysfunction or changes in expression of MKPs is a feature of a number of cancers [1], whilst roles in gluconeogenesis, insulin resistance and diabetes have also been established [2], [3]. Recent evidence also implicates a role in the regulation of immune responses [4]. Deletion of MKP-1 [5] and PAC-1 [6] have been shown to both enhance and reduce LPS mediated cellular responses respectively, whilst MKP-5 is thought to regulate adaptive immunity via effects upon T-cells [7]. Furthermore, one of the main anti-inflammatory effects of dexamethasone is attributed to the induction of MKP-1 and the subsequent inhibition of p38 MAP kinase [8].

MAP kinase phosphatase-2 (MKP-2) is a class I DUSP [9], induced by growth factors, hormones and stress agents such as hydrogen peroxide. It is nuclear located due to two nuclear location sequences [10] and dephosphorylates ERK and JNK *in vitro* [11] whilst being ineffective for p38 MAP kinase despite binding strongly to this kinase [12]. Cellular studies suggest the potential of cell type specificity as stable or conditional over-expression of MKP-2 selectively inhibits JNK in epithelial cells types [13], [14]. Although often described as a surrogate to MKP-1, MKP-2 has been demonstrated to have roles in cellular apoptosis and senescence [13], [15]. A number of recent indirect studies have also indirectly implicated MKP-2 in cancer [16]. However, the function of MKP-2 in the immune system remains uncharacterised due to the lack of suitable MKP-2 (DUSP-4) knockout mouse models.

In this study we examined the function of the MKP-2 (DUSP4) gene using a novel MKP-2 knockout mouse. In macrophages derived from MKP-2^−/−^ mice, we find that its deletion results in enhanced JNK and p38 MAP kinase activation but not, as expected, increased ERK phosphorylation. Increased IL-6, IL-12, TNFα and PGE_2_ production suggested that MKP-2 may modify the innate immune response in a manner similar to that observed in the MKP-1 deletion model. However, in contrast to these changes we observed marked reduction in inducible nitric oxide (iNOS) expression and enhanced arginase-1 activation indicative of an additional regulatory function. We found that that bone-marrow derived macrophages from MKP-2^−/−^ mice were more susceptible to infection with the intracellular parasite *Leishmania (L.) mexicana* than macrophages from their wild-type counterparts. The enhanced susceptibility of MKP-2^−/−^ macrophages was reversed using the arginase inhibitor nor-NOHA. Consequently following infection with *L. mexicana* MKP-2^−/−^ mice had limited ability to control lesion development and parasite growth. This was related to a significant down-regulation of specific Th1 activity in MKP-2 deficient mice. Taken together, our results demonstrate for the first time that MKP-2 plays a functional role in limiting immune responses associated with the macrophage lineage and indicates for the first time that MKP-2 is not a functionally redundant DUSP *in vivo*.

## Results

### MKP-2 deletion in macrophages

**Figure 1:**
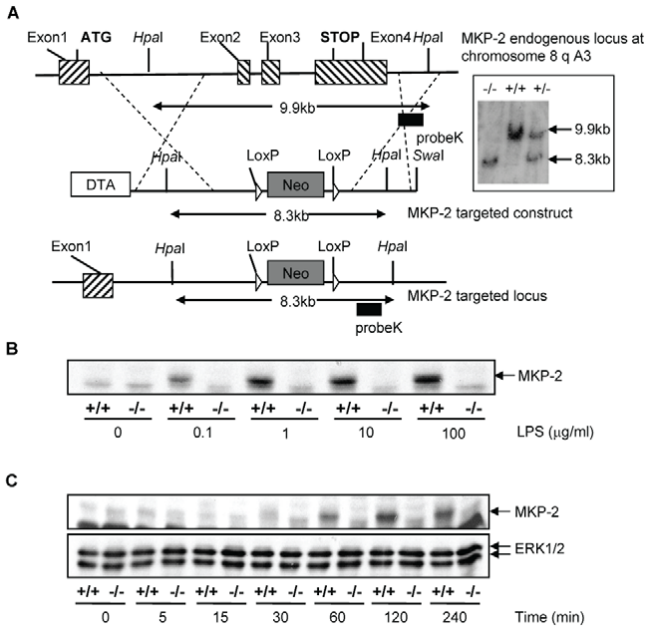
Generation of mice lacking DUSP4/MKP-2 gene by targeted homologous recombination. (Panel A): Schematic showing the DUSP4/MKP-2 gene locus, the targeted construct and the resulting targeted allele. Recombination events are indicated by dashed lines and show the replacement of an 8.3 kb *Swa*I DUSP4/MKP-2 genomic fragment containing exons 2–4 by the PGK-Neo cassette. *Swa*I and *Hpa*I described the restriction sites for the respective enzymes. The pGK-Neo cassette is flanked by LoxP sites. DTA represents the negative selection cassette. (Inset) An example of the 3′ southern blot analysis of mouse tail tip genomic DNA following digestion with *Hpa*I using an external Probe K as indicated in panel A. The autoradiography revealed the 9.9 kb (wild type) and 8.3 kb (targeted) fragments representing the two different alleles discriminating wild type, heterozygote or homozygote mutant animals. B & C: Concentration (LPS µg/ml for 60 min) and time dependent (LPS 100 ng/ml) expression of MKP-2 in bone marrow derived macrophages. The blot represents at least 4 individual experiments.

The MKP-2 deletion strategy is outlined in Figure 1A. Using targeted homologous recombination exons 2–4 were removed. Therefore, most of the open reading frame including the phosphatase catalytic domain and the 3′UTR region of the DUSP4 gene was deleted. Although exon1, which comprises the 5′UTR, the start codon and the KIM domain, remained unchanged, the occurrence of a truncated protein was unlikely since the poly A tail necessary for any kind of protein synthesis was removed. Deletion was confirmed by Southern Blotting (insert), which identified both the wild type 9.9 kB fragment and the mutated 8.3 kB fragment and by PCR (not shown). In Panels B & C, bone marrow derived macrophages were assessed for the presence of MKP-2 protein by Western blotting. In wild type cells, LPS stimulated MKP-2 expression in a concentration-dependent manner, giving a maximum response at approximately 1 µg/ml LPS (Panel B). Induction was also time dependent reaching a peak at 2 h following LPS treatment (Panel C). No induction was observed in macrophages derived from MKP-2^−/−^ mice.

### MKP-2 deletion enhances MAP kinase signalling

**Figure 2:**
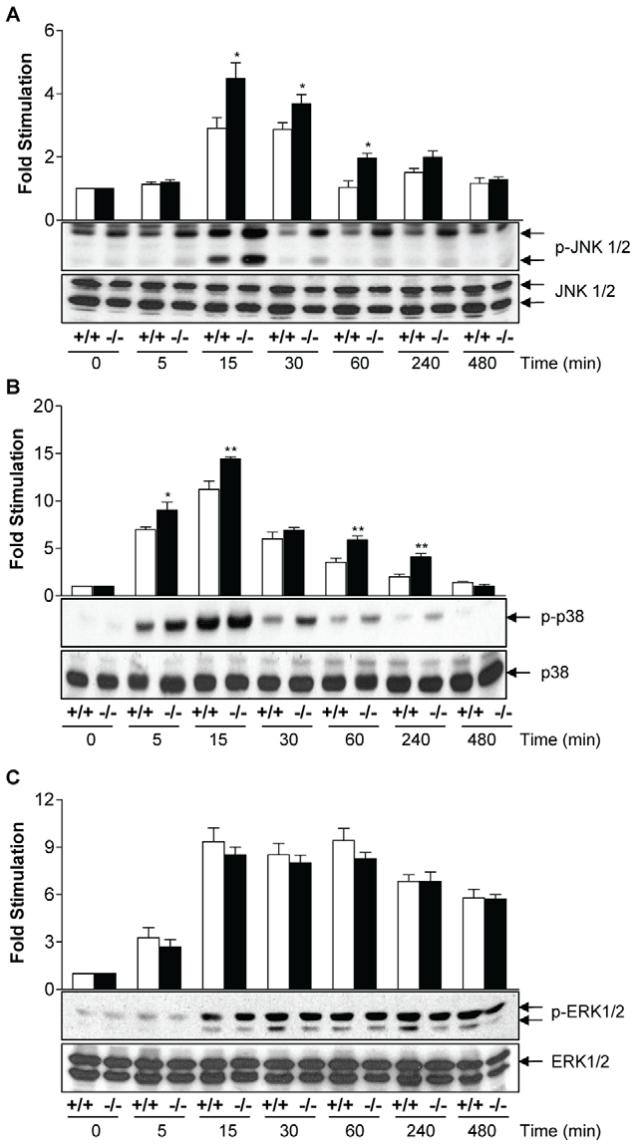
MKP-2 deletion enhances LPS -stimulated MAP kinase phosphorylation in macrophages. Cells were incubated with LPS (100 ng/ml) for the indicated time and whole cell lysates were prepared, and assessed for JNK (Panel A), p38 MAP kinase (Panel B) or ERK (Panel C) phosphorylation or total levels by Western blotting as outlined in the Methods section. Each blot is representative of 4 individual experiments. * P<0.05 relative to MKP-2^+/+^ macrophages.

We then examined the effect of MKP-2 deletion on LPS-induced kinase phosphorylation (Figure 2) as MKP-2 has previously been shown to dephosphorylate ERK and JNK *in vitro* [11]. In MKP-2^−/−^ macrophages, LPS-stimulated JNK phosphorylation was potentiated and prolonged (Panel A *p<0.05). Enhanced JNK activity was confirmed by JNK *in vitro* kinase assay and Western blotting for serine-63 phosphorylation of c-Jun (Supplementary Figure S1). Surprisingly, LPS-stimulated p38 MAP kinase phosphorylation (Panel B) was also found to be enhanced in MKP-2 ^−/−^ mice, despite p38 MAP kinase not being a recognised substrate for MKP-2 *in vitro* [11]. In contrast, ERK activation was not altered in MKP-2^−/−^ macrophages at any time point studied or over different concentration ranges (Panel C). Preliminary results, however, showed a lack of ERK translocation to the nucleus (results not shown) suggesting a lack of access of ERK to the phosphatase rather than a lack of MKP-2 activity on ERK as the explanation for this finding.

### Cytokine induction modified in MKP-2 knockout macrophages

**Figure 3:**
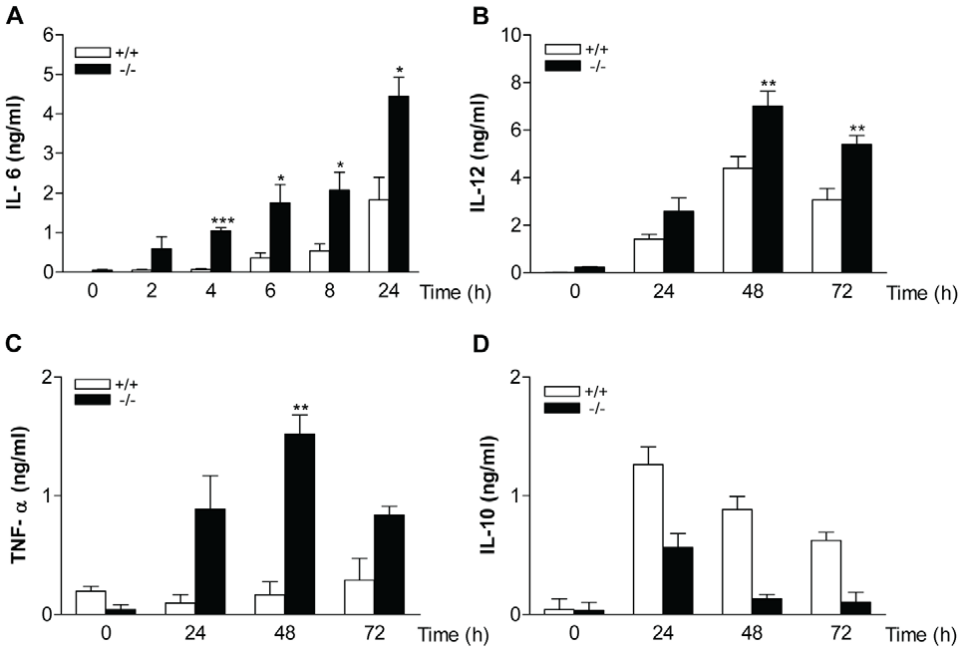
Cytokine production in macrophages derived from MKP-2^−/−^ mice. Cells were stimulated with LPS (100 ng/ml) for the indicated times and supernatants assessed for either IL-6 (panel A), IL-12 (Panel B), TNFα (Panel C) or IL-10 (Panel D) as outlined in the Methods section. Each value represents the mean ± SEM from at least 4 individual experiments. * P<0.05 compared to MKP-2 ^+/+^ macrophages.

Having established that enhanced kinase activation occurred in macrophages from MKP-2^−/−^ mice, we also assessed the consequences of MKP-2 deletion on the expression and release of key cytokines, known to be regulated by MAP kinase activation. Figure 3 shows cytokine production in macrophages derived from both wild type and MKP-2^−/−^ mice. Panel A shows that LPS induced a substantial increase in IL-6 production manifest at 6–8 h and reaching a peak after 24 h. MKP-2 deletion had little effect upon basal levels, however, but markedly enhanced the rate and magnitude of production stimulated by LPS (24 hr: MKP-2^+/+^ = 1.83±0.01 ng/ml, MKP-2^−/−^ = 4.44±0.83 n = 4, P<.005). Similar results were observed for both IL-12, which was assayed as IL-12p40/70 (Panel B), and TNFα (Panel C) although production reached a peak at 48 h and the degree of potentiation, whilst significant, was less than that for IL-6. In contrast, LPS stimulated IL-10 production was markedly reduced in MKP-2 deficient macrophages (Panel D).

### Reciprocal effects upon iNOS and arginase-1 following MKP-2 deletion

**Figure 4:**
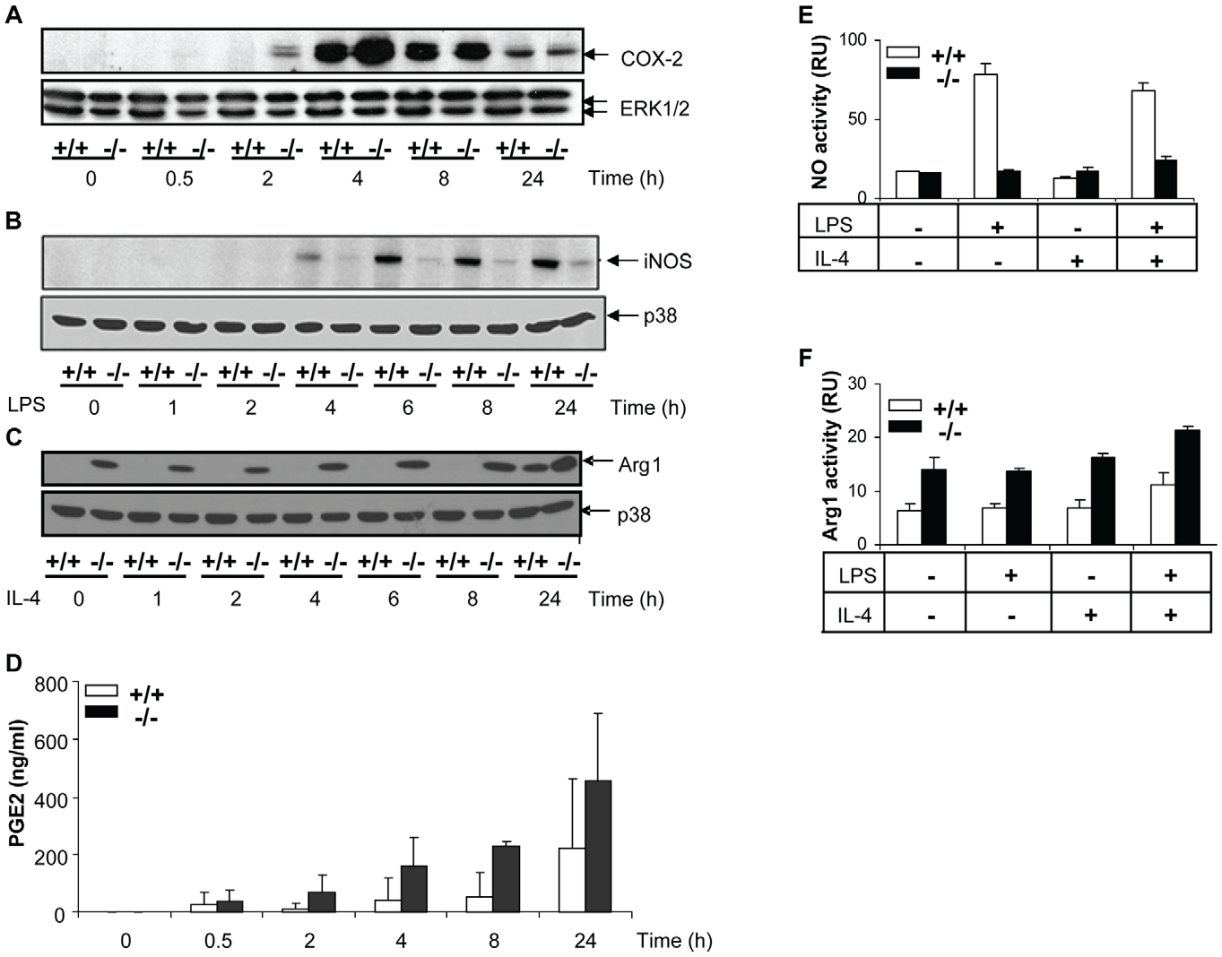
Differential effects of MKP-2 deletion upon COX-2, iNOS and Arginase-1 expression in LPS-stimulated macrophages. Cells were stimulated with LPS (100 ng/ml) for the indicated times and whole cell lysates were prepared, and assessed for COX-2 (Panel A), iNOS (Panel B) and Arginase-1 (Panel C) by Western blotting as outlined in Materials and Methods. Blot is representative of three individual experiments. Samples were assessed for associated PGE_2_ histogram (D), Nitrite (E) and arginase activity (F).

We then assessed the expression of a number of inflammatory proteins following MKP-2 deletion (Figure 4). Panel A shows that in MKP-2^−/−^ macrophages the rate of onset of cyclo-oxygenase-2 (COX-2) expression was increased in response to LPS, relative to wild type controls, an outcome predicted by previous studies assessing MAP kinase regulation of COX-2 expression and consistent with enhanced JNK and p38 MAP kinase activation. A difference in induction was observed as early as 2 and 4 h and was consistent with increased PGE_2_ production (Panel A and histogram D). We also assayed inducible nitric oxide synthase (iNOS) expression following incubation with LPS for up to 24 h. However, rather than being enhanced, iNOS expression was strongly inhibited relative to wild type controls and this was reflected in reduced formation of nitric oxide - derived nitrate and nitrite (Figure 4, Panel B and histogram E). This strong inhibitory effect was reproduced when IFN-γ was used to induce iNOS instead of LPS (Supplementary Figure S2). IFN-γ is known to regulate iNOS expression via the JAK/STAT pathway but no differences where found in the activation of these intermediates in MKP-2^+/+^ and MKP-2^−/−^ macrophages (Supplementary Figure S2). We also determined if other macrophage proteins were down regulated to a similar extent, specifically arginase-1, a protein stimulated through the alternative macrophage activation pathway and utilising the same substrate, L-arginine, as iNOS (Panel C). Basal arginase-1 expression and activity was considerably higher in MKP-2^−/−^ macrophages, and equivalent to 24 h of IL-4 stimulation in MKP-2^+/+^ samples (Panel C and histogram F). These results contrasted greatly with the expression of iNOS, which is indicative of classical macrophage activation and shows that the MKP-2^−/−^ macrophages intrinsically have a unique profile of inflammatory protein expression.

### MKP-2 deficiency results in increased susceptibility to *Leishmania mexicana* infection

Changes in MAP kinase signalling and iNOS activity are implicated in the ability of mice to resist infection with *Leishmania* [17], [18], [19], [20], [21], [22]. However, while the signalling and associated cytokine changes in MKP2^−/−^ mice would favour increased protection, the unexpected changes in iNOS and arginase expression would favour disease progression. As *L. mexicana* induces an intermediate disease phenotype in C57BL/6 and B6/129 mice whereby lesion growth is controlled, but fails to heal [23], we used this pathogen to test the ultimate influence of MKP-2 on disease outcome.

**Figure 5:**
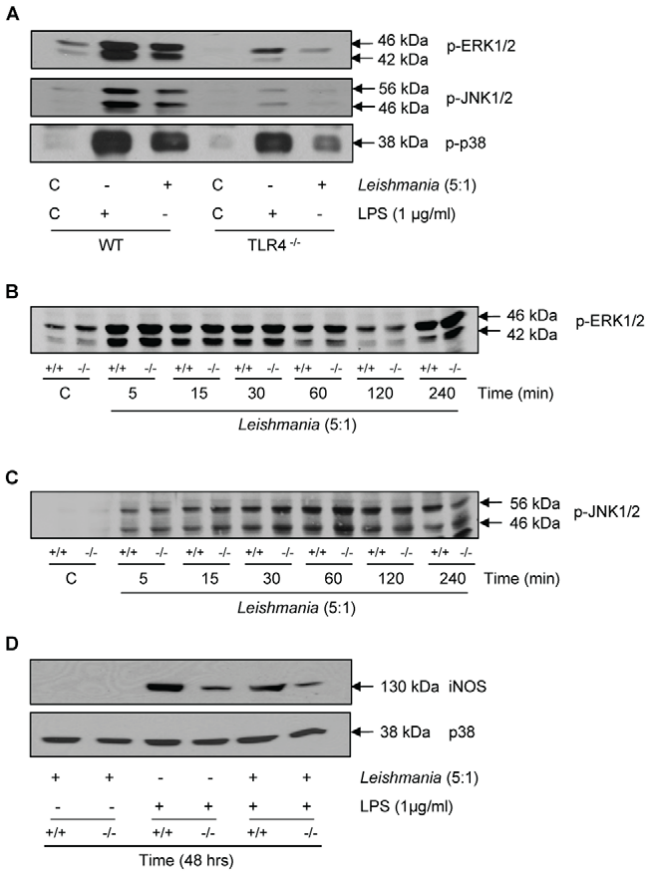
Macrophage activation by *Leishmania mexicana*. In Panel A macrophages from wild type (WT) and TLR-4 deletion mice (TLR-4^−/−^) were incubated with LPS (100 ng/ml) or promastigotes (5∶1) for 30 min. In panels B and C, MKP-2^+/+^ macrophages (+/+) and MKP-2^−/−^(−/−) were stimulated with promastigotes for the times indicated. In Panel D cells were incubated promatigotes alone or in combination with LPS for 48 h. Samples were assessed for ERK, JNK and p38 MAP kinase phosphorylation or iNOS expression as indicated. Each blot is representative of three individual experiments.

Initially, we tested the effect of *Leishmania mexicana* promastigotes on cellular MAP kinase signalling responses and iNOS induction (Figure 5) to confirm that both LPS, via TLR-4, and promastigotes mediate common kinase signalling cassettes and related functional end points. We initially found that in wild type macrophages, promastigotes activated ERK, JNK and p38 MAP kinase (Panel A). However, in TLR-4 deficient macrophages, both ERK and JNK phosphorylation were abolished in response to promastigotes, with a substantial reduction in p38 MAP kinase. In TLR-4^−/−^ macrophages the LPS-induced responses were also reduced, but this not by as much as the reduction seen in promastigote-induced responses. This is probably because our commercial source of LPS may have been contaminated with bacterial lipoproteins resulting in some activation of TLR-2. Nevertheless, these data suggest that both agents act principally through TLR-4. MKP-2 deletion had little effect upon the ERK response (Panel B) or p38 MAP kinase signalling (not shown) in response to promastigotes and JNK phosphorylation was only marginally increased although not significantly (Panel C). Despite activation of these kinases, promastigotes alone failed to significantly induce MKP-2 protein (data not shown) and had no effect upon iNOS synthesis alone or in response to LPS (Panel D). Furthermore, promastigotes did not significantly increase the already high levels of arginase-1 activity in MKP-2^−/−^ macrophages (arginase activity, µg/ml: MKP-2^+/+^ control = 125.4±14.3, *L*. *mexicana* = 101.1±13.0; MKP-2^−/−^ control = 345.8±17.10, *L. mexicana* = 392±23.5 n = 3).

**Figure 6:**
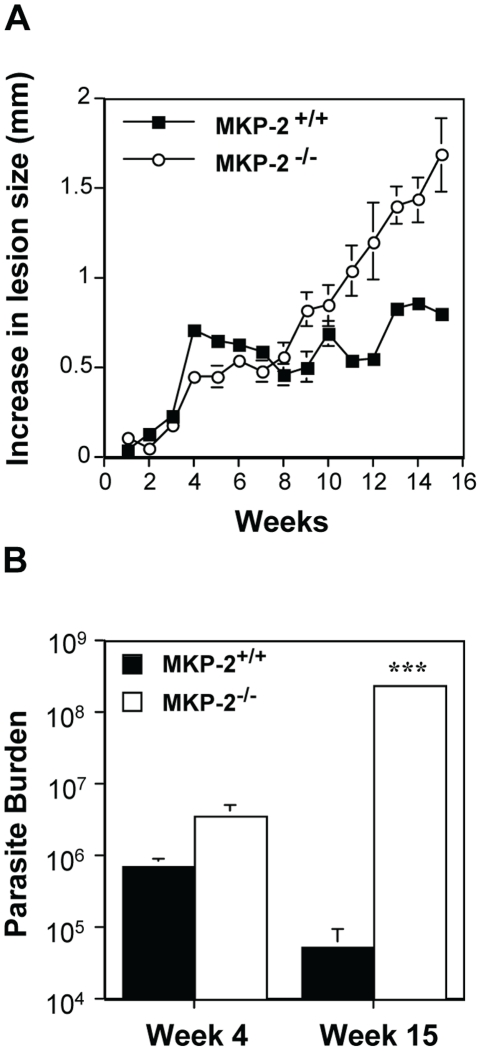
MKP-2 deficiency increases susceptibility to *L. mexicana* infection in the footpad. Infection was with 5×10^6^ *L. mexicana* stationary phase promastigotes into the footpad and susceptibility was measured by mean ± SEM lesion growth (Panel A), and parasite burdens (***p<0.001) (Panel B). MKP-2^−/−^ (n = 8), unlike MKP-2^+/+^ mice (n = 7) were unable to control lesion growth (Panel A) and this was reflected in the comparative parasite burdens (Panel B) in the footpad. Representative results from 3 similar experiments.

**Figure 7:**
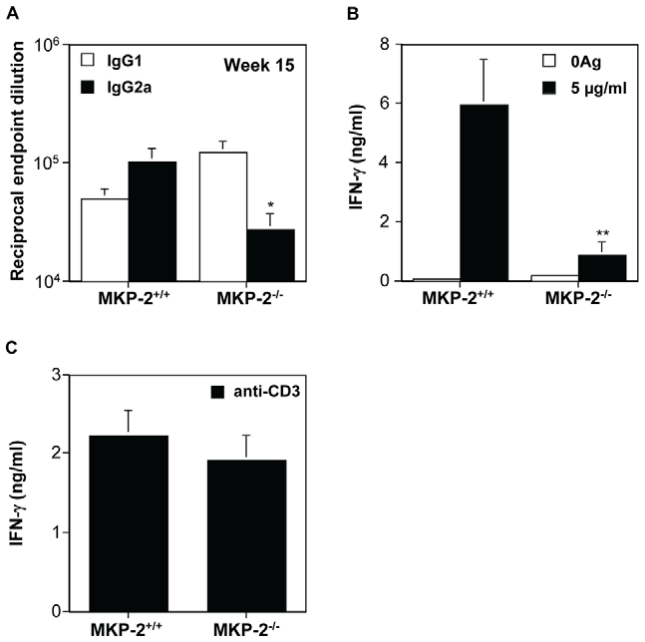
MKP-2 deficiency results in a limited Th1 response following footpad infection with *L. mexicana*. Th1 responses were measured by specific serum IgG2a antibody (*p<0.05) (Panel A), and antigen induced splenocyte IFN-γ production (**p<0.01) (Panel B).Results are expressed as mean ± SEM of MKP-2^−/−^ (n = 8) and MKP-2^+/+^ (n = 7) mice. MKP-2 deficiency did not directly impair the ability of T cells to mount a Th1 response as anti-CD3 stimulation of splenocytes from both infected and non-infected MKP-2^+/+^ and MKP-2^−/−^ mice induced similar IFN-γ production (Panel C). Representative results from 3 similar experiments.

Nevertheless, despite promastigotes being unable to modify MKP-2 expression *per se*, we found that MKP-2 deletion had a significant effect *in vivo* following *Leishmania mexicana* infection. Upon injection into the footpad with *L. mexicana*, MKP-2^−/−^ mice developed progressively growing lesions and could not limit lesion growth unlike their wild-type counterparts (Figure 6A). Lesions grew more rapidly in MKP-2^+/+^ mice compared with MKP-2^−/−^ mice in the first 4 weeks of infection but the parasite burdens remained higher at this site in MKP-2^−/−^ mice than their wild-type counterparts throughout infection (Figure 6B). At week 15, the Th1 response, as measured by antigen specific IgG2a and IFN-γ production (Figures 7A and B), was significantly reduced in MKP-2^−/−^ compared with MKP-2^+/+^ mice confirming that the MKP-2^−/−^ mice were defective in their ability to control parasite growth. No differences in the Th2 response were noted with specific IgG1 (Figure 7A) and whole IgE as well as IL-4, IL-13, and IL-10 production (data not shown), all being similar in MKP-2^−/−^ and MKP-2^+/+^ mice. There was no evidence of an intrinsic T cell defect in MKP-2^−/−^ mice as splenocytes from infected MKP-2^−/−^ and MKP-2^+/+^ mice produced similar levels of IFN-γ upon stimulation with anti-CD3 (Figure 7C).

**Figure 8:**
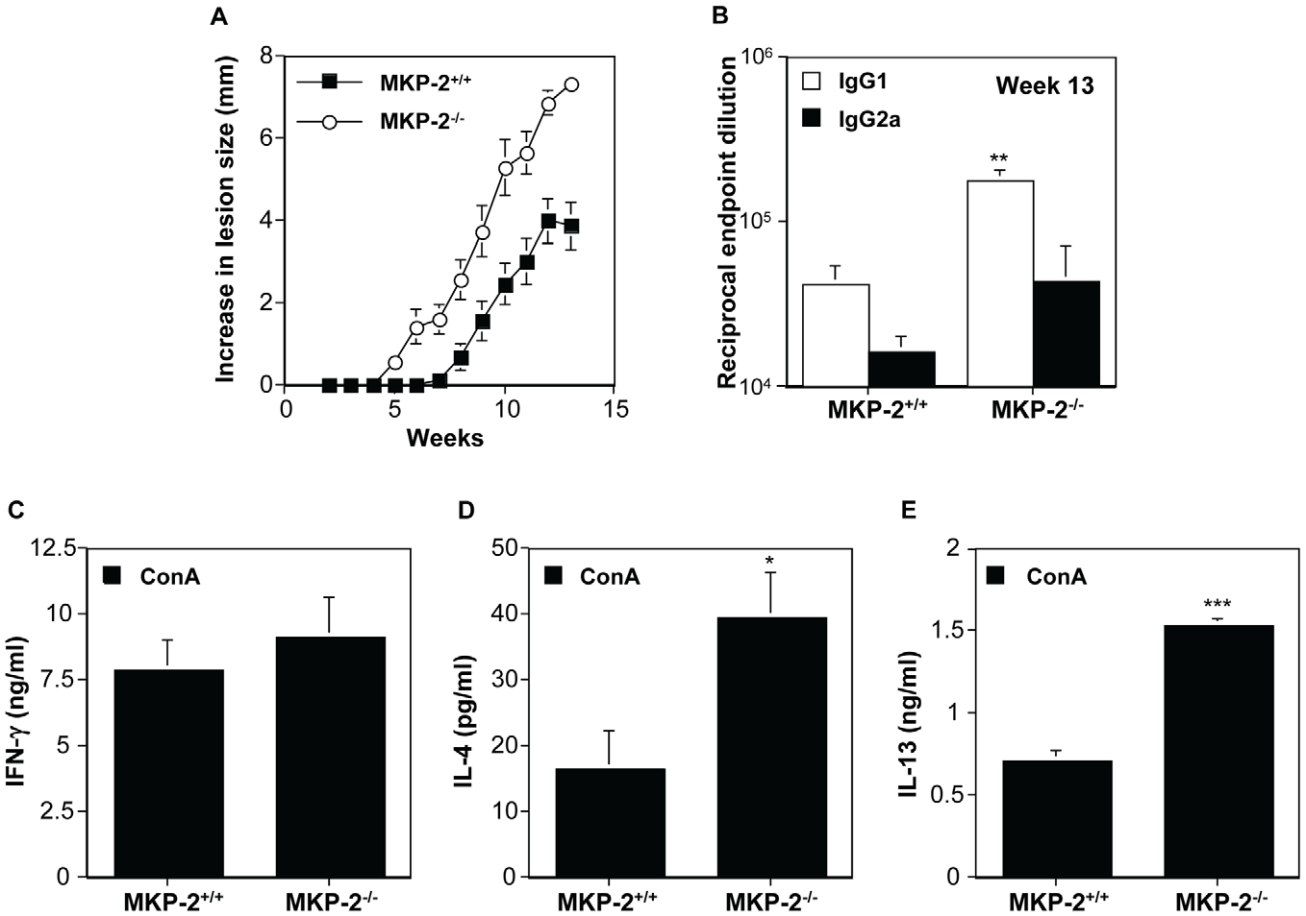
MKP-2 deficiency results in increased susceptibility and an enhanced Th2 response following infection in the rump with *L. mexicana*. Lesions developed quicker and were of greater size in MKP-2^−/−^ (n = 6) mice compared with MKP-2^+/+^ (n = 6) mice (Panel A). Enhanced Th2 responses in MKP-2^−/−^ mice were measured by increased specific serum IgG1 antibody production compared with wild-type counterparts (**p<0.01) (Panel B). In addition while ConA induced splenocyte IFN-γ production was similar in MKP2^−/−^ and MKP-2^+/+^ mice (Panel C), IL-4 (*p<0.05) (Panel D) and IL-13 (***p<0.001) (Panel E) levels were significantly elevated in MKP-2^−/−^ animals. Results are expressed as mean ± SEM of MKP-2^−/−^ and MKP-2^+/+^ mice.

The growth of *L. mexicana* is subject to different immunological controls at different sites. The non-healing response to *L. mexicana* in infected footpads is associated with deficient IFN-γ production, independent of a Th2 response, whereas in the back rump the host response to infection is entirely Th2 dependent [24]. Consequently, we monitored the growth of *L. mexicana* in the shaven back rump of MKP-2^−/−^ and MKP-2^+/+^ mice (Figure 8). Lesions appeared earlier and lesion growth was more rapid in MKP-2^−/−^ mice (Figure 8A). The increased susceptibility of MKP-2^−/−^ mice over wild-type animals was associated with expanded Th2 responses in these animals as measured by significantly increased specific IgG1 production (Figure 8B) and increased ConA induced splenocyte IL-4 (Figure 8D) and IL-13 (Figure 8E) production. At the same time there were no significant differences between infected MKP-2^−/−^ and MKP-2^+/+^ in IFN-γ production (Figure 8C) and specific IgG2a levels (Figure 8B). As with footpad infections there was no indication of an intrinsic T cell defect as measured by anti-CD3 splenocyte cytokine production (results not shown).

### MKP-2 deficient bone marrow-derived macrophages have impaired ability to control the growth of *L. mexicana*

**Figure 9:**
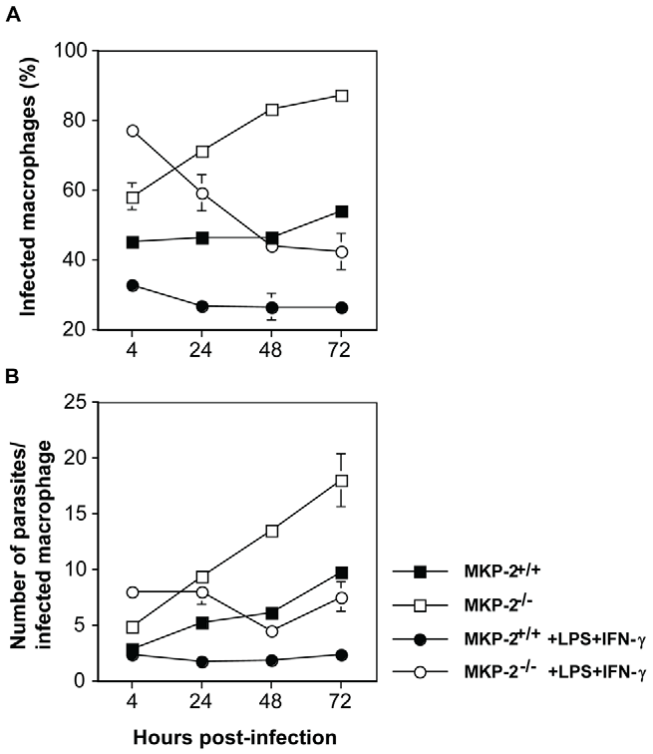
MKP-2 deficiency results in impaired ability of macrophages to control *L. mexicana* infection. Both the percentage of infected macrophages (p<0.005) (Panel A) and the numbers of parasites/infected macrophage (p<0.01) (Panel B) were significantly greater in non-stimulated bone marrow derived-macrophages from MKP2^−/−^ donors compared with MKP-2^+/+^ mice by 72 h post-infection. While stimulation with 100 ng LPS + 100U IFN-γ significantly inhibited both the percentage (Panel A) of infected macrophages and the mean number of parasites/infected macrophage (Panel B) in the cultures derived from MKP-2^−/−^ mice compared with non-stimulated MKP-2^−/−^ macrophages this was only to levels comparable with those found in non-stimulated macrophages from MKP-2^+/+^ mice at 72 h post-infection. Each figure represents 3 experiments (mean ± SEM of triplicate determinations).

To confirm that the intrinsic defect in infectivity was at the level of the host macrophage we therefore compared the growth of *L. mexicana* parasites in MKP-2^−/−^ and MKP-2^+/+^ bone marrow derived macrophages. Macrophages were infected with promastigotes at a multiplicity of infection (M.O.I) of 5 parasites/macrophage and growth monitored at 4, 24, 48 and 72 h post-infection in resting as well as LPS+IFN- γ stimulated macrophages (Figure 9). Macrophages from MKP-2^−/−^ mice were significantly more permissive to infection than MKP-2^+/+^ macrophages as measured by the percentage of cells infected by 4 h (p<0.01) (Figure 9A). Similarly, parasite growth was significantly enhanced in MKP2^−/−^ macrophages compared with MKP-2^+/+^ macrophages under non-stimulated conditions up to 72 h post-infection (Figure 9B). MKP-2^−/−^ macrophages were, however, able to control parasite growth following IFN- γ+LPS stimulation although only to a level comparable with non-stimulated macrophages derived from MKP-2^+/+^ bone marrow. This would be consistent with data in figure 5 showing that after 48 h there is measurable iNOS expressed in MKP-2^−/−^ KO macrophages in response to LPS although much less than in MKP-2^+/+^ macrophages.

### L-arginine inhibition reverses the effect of MKP-2 deficiency on parasite growth

**Figure 10:**
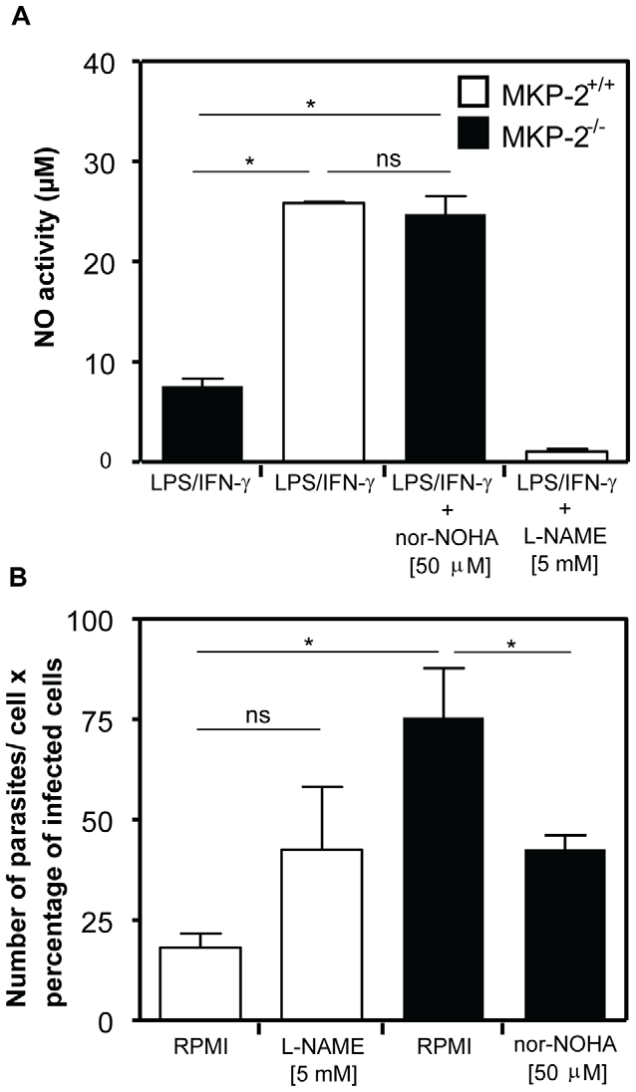
Arginase inhibition reverses the susceptibility of MKP-2^−/−^ macrophages to control *L. mexicana* infection. Macrophages were pre-treated with or without inhibitors for 60 min as indicated prior to activation with LPS (100 ng/ml) plus IFN-γ (100 U/ml) and samples assayed for NO after 48 hr (Panel A). In panel B following treatment with inhibitors macrophages were incubated with *L*. *mexicana* promastigotes (5∶1) and survival measured after 48 h as outlined in the Material and Methods section. Data is displayed as a composite of the number of parasites per cell and the percentage of infected cells.Results are expressed as mean ± SEM. *P<0.05 compared to vehicle treated cells (RPMI).

Figure 10 shows the effect upon NO production and parasite infection following the treatment with the arginase inhibitor N^ω^-hydroxy-nor-Arginine (nor-NOHA). Initially, we assessed NO release to confirm that arginase inhibition had been effective in altering, as predicted, the levels of NO in macrophages (Panel A). In LPS+IFN-γ -stimulated MKP-2^−/−^ macrophages NO levels were, as predicted, low, however following nor-NOHA treatment, levels increased significantly equivalent to the level observed for wild type macrophages. In addition, treatment of MKP-2^+/+^ macrophages with nor-NOHA failed to significantly increase the levels of NO further. When assessing parasite growth in MKP-2^−/−^ and MKP-2^+/+^ macrophages following nor-NOHA pre-treatment we found that changes in arginase activity altered infectivity (Panel B). In MKP-2^−/−^ macrophages, infectivity was high relative to wild type as expected. However, infectivity was significantly reduced in response to nor-NOHA, to a level which was not significantly different from MKP-2^+/+^ macrophages. Inhibition of NO using L-NAME also increased the infectivity of the parasite in MKP-2^+/+^ macrophages suggesting NO is a determinant of macrophage resistance to infectivity with *L. Mexicana* promastigotes. Furthermore, L-NAME treatment of infected MKP-2^−/−^ macrophages resulted in rapid macrophage destruction and release of parasites (>90%) making it impossible to quantify changes in infectivity. This finding nevertheless re-enforced the idea of increased sensitivity of MKP-2^−/−^ macrophages to infection *per s*e.

## Discussion

This study demonstrates for the first time a novel immune function for MKP-2 *in vivo* primarily caused by changes in the ability of macrophages to induce innate immune responses. In particular, MKP-2 deletion gives rise to a novel phenotype associated with decreased iNOS and increased arginase-1 activity which makes MKP-2 deficient macrophages more intrinsically susceptible to infection with the intracellular parasite *L. mexicana*. Infection of MKP-2 deficient mice not only results in increased disease susceptibility and parasite growth *in vivo* compared to their wild-type counterparts but it is also associated with either inhibition of a Th1 response or promotion of a Th2 response appropriate to enhance infection at the site utilised.

In characterising the novel MKP-2^−/−^ model, we clearly demonstrate that following gene deletion JNK phosphorylation and activity was enhanced. This is consistent with the original studies *in vitro* [11] and more recent studies using either stable or conditional expression of MKP-2 [13], [14]. ERK activation was, however, not enhanced in MKP-2^−/−^ macrophages despite being a substrate for MKP-2, due to compartmentalisation of ERK to the cytosol, generating a substrate selective function for MKP-2 in this cell type. Unexpectedly, we found that phosphorylation of p38 MAP kinase was also enhanced despite this kinase not being susceptible to dephosphorylation by MKP-2 *in vitro*. A recent cellular study, however, has indirectly implicated involvement of MKP-2 in regulating AMP kinase and p38 MAP kinase-dependent gluconeogenesis [25] and our findings support this data. Our data show that predictions based on *in vitro* data can be misleading and indeed study of other MKP deletion mice also shows discordance between substrate specificity *in vitro* and kinase regulation in whole cells. Whilst MKP-1 is effective against all three major MAP kinases, enhanced p38 MAP kinase is observed in MKP-1^−/−^ macrophages [26], whilst in cells derived from PAC-1 knockout mice, ERK phosphorylation is inhibited despite ERK being a specific substrate for PAC-1 *in vitro* [6].

Our studies demonstrated that enhanced JNK and p38 MAP kinase activation where reflected at the level of cytokine synthesis. Evidence strongly supports a role for p38 MAP kinase in the expression of IL-6, TNFα and IL-12 [27], [28], [29]. In contrast, a role for JNK is less well defined and cell type specific involvement is implicated [27], [29]. Our studies also correlate well with those obtained with the MKP-1 deletion which implicates p38 MAP kinase as the main *in vivo* substrate for MKP-1 and shows this kinase to be linked to cytokine release [26], [30]. This would suggest that MKP-2 deletion, in a manner similar to MKP-1 [26], [30] could enhance innate immune responses *in vitro* and also possibly *in vivo*.

This possibility was contradicted by other findings. Whilst we found up regulation of COX-2 and associated PGE_2_ in MKP-2 deletion mice, results also observed in MKP-1 deletion mice, surprisingly expression of iNOS was ablated. Studies show that iNOS expression is enhanced following MKP-1 deletion *in vitro* [31] and associated with increased mortality *in vivo* [32]. This suggests the potential for MKP-2 deletion, unlike ablation of MKP-1, to protect against NO induced mortality. Furthermore, we find that basal arginase 1 expression and associated arginase activity is markedly increased in MKP-2 deletion mice, a response which would also mediate a reduction in NO formation, this is again different to the findings in MKP-1^−/−^ macrophages [31]. The molecular mechanisms underpinning the reciprocal regulation of these two inflammatory proteins are at present unclear, as none of the cognate signalling pathways regulating iNOS or arginase induction were found to be negatively affected. Thus, there is a potential for a direct action of MKP-2 within the nucleus which may directly regulate transcription. Recently, MKP-1 has been implicated in the regulation of histone phosphorylation in the nucleus [33] suggesting nuclear functions for the MKPs other than direct MAP kinase regulation.

Overall our studies *in vitro* therefore reveal that in the absence of MKP-2 macrophages take on a functional profile which can be directed towards either a type-1 or type-2 phenotype. Our *in vivo* studies enabled us to demonstrate which of these profiles predominate *in vivo*. *Leishmania* infection has a well recognized ability to subvert the development of Th1 responses partly via effects upon MAP kinase signalling [17], [20], [34]. Most of these studies have implicated MAP kinase involvement at the level of the host macrophage or dendritic cell, but JNK activation via T cells has also been shown to negatively regulate Th2 cells [34] in a healing response. Therefore, we hypothesised, given the enhanced p38 and JNK activation of MPK-2^−/−^ mice on the C57BL/6 background, that such animals would develop a healing phenotype against this organism. MKP-2 negatively regulated IL-12, IL-6 and TNF-α expression and positively regulates IL-10 confirming this hypothesis. Currently iNOS induction and the subsequent release of NO is considered protective against intracellular infection with many organisms including *Leishmania* species [19]. In addition, arginase-1, which competes for the same substrate as iNOS, has been shown to promote disease progression not only against *Leishmania* (in both healing and non-healing strains of mice [18]), but also *Mycobacterium* and *Toxoplasma gondii* [35]. As MKP-2 deficiency downregulates iNOS, but upregulates arginase-1 expression and activity this suggests that, counter intuitively, MKP-2^−/−^ animals would be more susceptible to *Leishmania* infection.

Our studies indeed show that MKP2^−/−^ mice are, in fact, more susceptible to infection with *L. mexicana* and it is the consequence of changes in iNOS and arginase rather than changes in kinase mediated inflammatory cytokine signalling which dictates the subsequent *in vivo* response to MKP-2 deletion. The differential effects of *Leishmania* infection in MKP-2^−/−^ macrophages relative to MKP-2^+/+^ is not due to *Leishmania* interacting with the cell in a different way to LPS, both agents utilised TLR-4 during the early stages of stimulation. Furthermore, the fact that *Leishmania mexicana* alone does not induce MKP-2 points to another potentially indirect effect of MKP-2 deletion not linked to changes in kinase activity. Nevertheless, following footpad infection with *L. mexicana*, lesion growth was increased and parasite burden enhanced, outcomes associated with reduced IFN-γ production. Of significance it has recently been demonstrated that during *L. major* infections high local arginase levels at the site of infection mediate L-arginine depletion, which results in impaired local CD4^+^ T cell function particularly IFN-γ production [21], [22]. The importance of arginase in modulating the virulence of *L. mexicana* is also highlighted by the fact that arginase null-mutant *L. mexicana* has attenuated virulence *in vitro* and *in vivo* suggesting that the parasite arginase depletes host L-arginine available for iNOS activity [36], [37]. Significantly, mice infected in the footpad with arginase null-mutant *L. mexicana* have increased antigen induced IFN- γ production. Thus changes in arginase-1 expression in MKP-2^−/−^ macrophage can be easily linked to observed changes in infectivity and immune responses.

Our study is one of the first to reveal an *in vivo* function for MKP-2 and indicates that MKP-2 does not act as a surrogate to the more extensively studied MKP-1. Our results reveal the potential of MKP-2 to participate in opposing regulatory mechanisms. The first mechanism is based on upregulation of JNK and p38 MAP kinase signalling and is associated with enhanced cytokine expression. The second regulatory mechanism however, involves changes in iNOS and arginase-1 expression, associated with the alternative activated macrophage pathway, is not readily associated with modulation in kinase signalling but possibly involves a different molecular target. The *in vivo* consequences of this second action can be clearly seen during *Leishmania* infection where there is down-regulation of Th1 and/or up-regulation of Th2 responses, distinguishing MKP-2 from any of the other MKPs believed to play a role in immune function.

## Materials and Methods

### Ethics statement

All animal procedures conformed to guidelines from The Home Office of the UK government. All work was covered by two Home Office licences: PPL60/3929, “mechanism of control of parasite infection” and PPL60/3439, “genetic models of cancer and inflammation”.

### Materials

Dulbecco's modified Eagle's medium and foetal calf serum (FCS) were purchased from Invitrogen. RPMI 164 and LPS were from Sigma Aldrich. Antibodies against p-ERK, and MKP-2 were obtained from Santa Cruz. All other phospho-antibodies were purchased from Biosource International Inc. (USA) and secondary antibodies from Jackson Immuno Research Laboratories Inc (PA, USA). The TLR-4 deficient mice were obtained from Professor *Akira S*. Osaka University, Japan.

### Methods

#### Generation of MKP-2 deficient mice

The deletion of the MKP-2 gene was performed in collaboration with Genoway, Lyon, France using standard procedures. The short arm and the long arm flanking both side of the cluster of exon 2–4 of the mouse MKP-2 gene was obtained by PCR using the following primers: for the small homology arm: 5′-GTGCCTGGTTCTGTGTGTGTCTGTTCTCC-3′ for the forward primer and 5′-TCTTACAGCCCTCTTTCCTCACGGTCG-3′ for the reverse primer producing a PCR fragment of 3009 bp. For the long homology arm: 5′-CTTTAGGAGCGACGGCCAGGAACACAGG-3′for the forward primer and 5′-ACCCTGCCACACAGGTTGGAGCAAGG-3′ for the reverse primer producing a PCR fragment of 6336 bp. Both arms were introduced into a PBS vector in either side of the neomycin cassette. The final vector was transfected into 129Sv mouse embryonic stem cell. Selection was made to select only the clones which had homologous recombination events first by PCR and then using Southern blotting for the short and the long arm on the construct. Male chimeras were obtained and crossed with C57Bl/6 female to obtain the F1 generation. The F1 generation was screened for germ line transmission of the mutation. Heterozygous mice were backcrossed against C57Bl/6 and genotyped using Southern blotting and PCR.

#### Generation of mouse macrophages

Bone marrow derived macrophages were isolated from femurs of 3 months old MKP-2^−/−^or ^+/+^ mice and grown in DMEM, containing 20% (v/v) heat-inactivated FCS supplemented and 30% L cell-conditioned medium supplemented with 5 mM L-Glutamine, 100 U/ml penicillin, 1 µg/ml streptomycin. Adherent cells were harvested and then seeded in either 12 well (1×10^6^ cells/ml) or 96 well (2×10^6^ or 2×10^5^ cells/ml) plates with RPMI 1641 supplemented with 10% FCS.

#### Western blotting

Proteins (15 µg per lane) were separated by 10% SDS-PAGE and transferred onto nitrocellulose. The membranes were blocked for non-specific binding for 2 h in 2% BSA (w/v) diluted in NATT buffer 50 mM Tris-HCl, 150 mM NaCl, 0.2% (v/v) Tween-20. The blots were then incubated overnight with 50 ng/ml primary antibody diluted in 0.2% BSA (w/v) in NATT buffer. The blots were washed with NATT buffer for 90 min and incubated with HRP-conjugated secondary antibody (20 ng/ml in 0.2% BSA (w/v) diluted in NaTT buffer) for 2 h. After a further 90 min wash, the blots were subjected to ECL reagent and exposed to Kodak X-ray film.

#### *Leishmania mexicana* parasites and infection model

*L. mexicana* (MYNC/BZ/62/M379) was maintained by serial passage of amastigotes inoculated into the shaven rumps of BALB/c mice. Infections were initiated using stationary phase promastigotes grown in TC100 insect medium (Sigma, St. Louis, USA) supplemented with v/v 10% FCS (Harlan Sera-Lab Ltd., Crawley). Promastigotes (5×10^6^ in 25 µl) were inoculated subcutaneously into the hind footpad or subcutaneously into the shaven back rump. Female 6–8 week old MKP-2^−/−^ and MKP-2^+/+^ mice were used in each experiment. Increase in footpad size was measured using a dial gauge micrometer and rumps with a slide gauge micrometer at weekly intervals. Parasites were enumerated from lesions as previously described [23].

#### Macrophage infection with *L. mexicana promastigotes*

One hundred microlitres of bone marrow-derived MKP-2^−/−^ or MKP-2^+/+^ macrophages (2×10^6^/ml) in RPMI 1641 supplemented with 10% FCS were added to each well of a 24-well tissue culture plate (Techno Plastic Products, Trasadingen, Switzerland) containing round 13-mm cover slips. Cells were infected by adding 100 µl *L. mexicana* stationary phase promastigotes at a parasite: host cell ratio of 5: 1 and incubating the cells for 4 h at 33°C The medium was then changed to remove unattached parasites and replenished with 100 µl fresh medium or medium containing IFN-γ or LPS alone or in combination. Cells were fixed in methanol (Banford Laboratories, Norden Rochdale, UK) and stained with 10% (v/v) aqueous Giemsa stain (BDH Laboratory Supplies) at 4, 24, 48 and 72 h post infection so that the percentage of cells infected and the number of parasites/100 infected macrophages could be determined by microscopy. To inhibit arginase or iNOS activity, cells were pre-treated for 1 h with 50 µM N^ω^-hydroxy-nor-Arginine (nor-NOHA, Calbiochem) or 5 mM N (G)-nitro-L- arginine methyl ester (L-NAME, Sigma), respectively. *L. mexicana* promastigotes were added at an M.O.I. of 5 and plates were incubated at 34°C. After 48 h, medium was removed, cells fixed with methanol and subsequently stained with Giemsa. Coverslips were mounted onto microscopic glass slides and parasite number and infection rate for a total of 200 macrophages per coverslip was assessed using a bright field microscope.

#### Immunological analysis

ELISA was routinely used for detection of IL-6, IL-12, TNFα and PGE_2_ in macrophages and *L. mexicana* specific-IgG1 and -IgG2a and total IgE in the plasma of infected mice as previously described [38]. Splenocytes were cultured in 96-well plates as previously described [39] and IFN-γ, IL-10, IL-13 and IL-4 production measured by capture ELISA.

#### Measurement of arginase activity

Arginase activity was measured using an assay based on a reaction with α-isonitrosopropiophenon (ISPF). Briefly, macrophages were grown on 24- well plates, exposed to agonist as appropriate and harvested in 50 µl lysis buffer (50 mM Tris-HCl, 10 mM MnCl_2_, 0·1% Triton X-100, 5 µg/ml pepstatin A, 5 µg/ml aprotinin, and 5 µg/ml antipain hydrochloride, pH 7.4). Arginine hydrolysis was carried out by incubating cell lysates with 25 µl of 0·5 M L-arginine (pH 9.7) at 37°C for 60 min. The reaction was terminated by adding 400 µL of an acid solution (H_2_SO_4_, H_3_PO_4_ and H_2_O in a ratio of 1∶3∶7 and 25 µL of a 9% solution of ISPF). Samples along with known urea standards were incubated at 95°C for 45 minutes, and then allowed to cool for 10 min in the dark. Aliquots were added to wells of a 96 well plate and absorbance read at 540 nm on a Spectromax 190 plate reader.

#### NO measurement in cell culture supernatants

Cell culture medium supernatant was collected and nitrite was analysed as a measure for NO production using the Griess reagent. Equal volumes of Griess reagent (1% (w/v) sulphanilamide/ 0.1% (w/v) N-(1-naphtyl)ethylenediamine dihydrochloride/ 2.5% (v/v) H_3_PO_4_) and cell culture supernatant were mixed and incubated at room temperature in the dark for 10 min. Absorbance was read at 540 nm on a Spectromax 190 plate reader. Nitrite production was determined using NaNO_2_ as standard.

#### Statistical analysis

Statistical significance was determined between groups using the Mann-Whitney U test for endpoint antibody titrations, as well as Student's t-test and one-way ANOVA with Dunnett's post test where appropriate. All experiments were performed at least three times with similar findings.

## Supporting Information

Figure S1 MKP-2 deletion enhances LPS-stimulated JNK activation and c-Jun phosphorylation in mouse bone marrow macrophages. Macrophages from MKP-2^+/+^ (open bars) and MKP-2^−/−^ (closed bars) mice were incubated with LPS (100ng/ml) for the indicated times and whole cell lysates prepared and assessed for JNK activity by *in vitro* kinase assay (Panel A) and p-c-Jun and c-Jun content (Panel B) by Western blotting. Each blot is representative of at three individual experiments. Each quantified value is expressed as mean ± SEM.
(3.44 MB TIF)

Figure S2 IFNγ -stimulated iNOS induction is severely ablated in MKP-2 deficient macrophages but not due to JAK/STAT pathway disruption. Macrophages from MKP-2^+/+^ (+/+) or MKP-2^−/−^ (−/−) mice were challenged with IFN-γ for the indicated times. iNOS expression (Panel A) and phosphorylation of JAK (Panel B) and STAT (Panel C) were assessed by Western blotting. Each blot is representative of at three individual experiments. Each quantified value is expressed as mean ± SEM.
(4.86 MB TIF)
