# Supplementary figures and images for: MAP Kinase Phosphatase-2 Plays a Critical Role in Response to Infection by Leishmania mexicana
Source: PLoS Pathog. 2010 Nov 11;6(11):e1001192. doi: 10.1371/journal.ppat.1001192 (PMC2978729; doi:10.1371/journal.ppat.1001192)

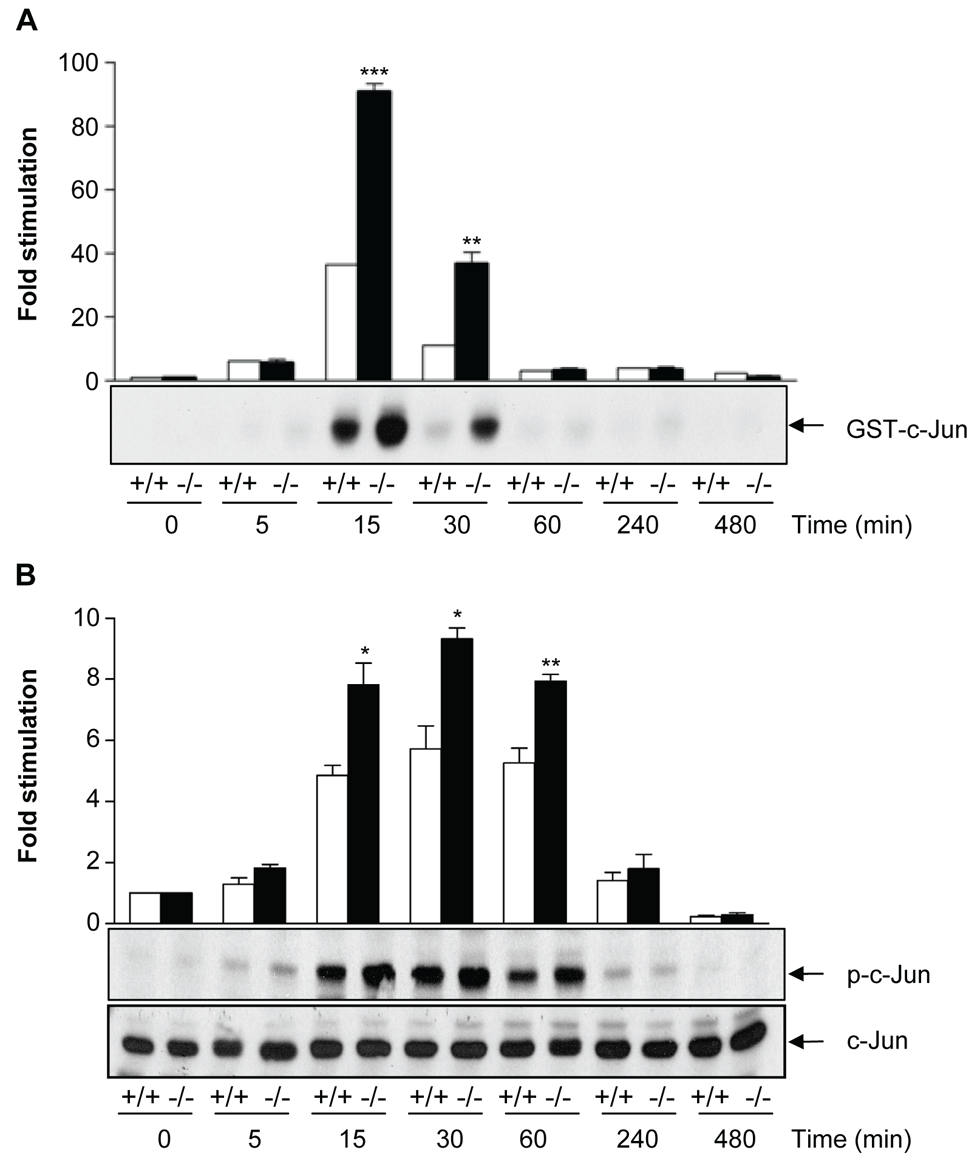

Supplement: Figure S1 — MKP-2 deletion enhances LPS-stimulated JNK activation and c-Jun phosphorylation in mouse bone marrow macrophages. Macrophages from MKP-2+/+ (open bars) and MKP-2−/− (closed bars) mice were incubated with LPS (100ng/ml) for the indicated times and whole cell lysates prepared and assessed for JNK activity by in vitro kinase assay (Panel A) and p-c-Jun and c-Jun content (Panel B) by Western blotting. Each blot is representative of at three individual experiments. Each quantified value is expressed as mean ± SEM. (3.44 MB TIF) [file ppat.1001192.s001.tif]

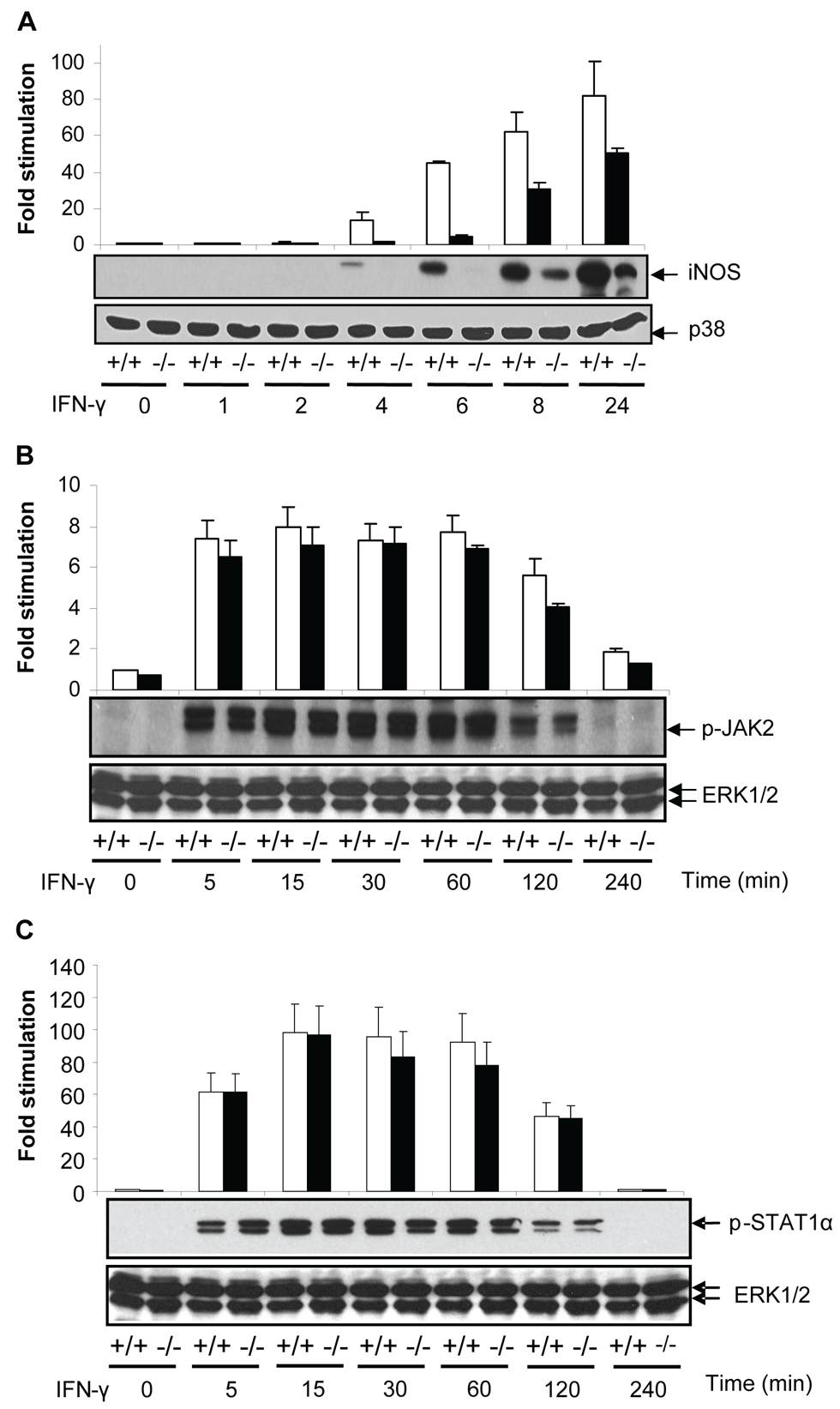

Supplement: Figure S2 — IFNγ -stimulated iNOS induction is severely ablated in MKP-2 deficient macrophages but not due to JAK/STAT pathway disruption. Macrophages from MKP-2+/+ (+/+) or MKP-2−/− (−/−) mice were challenged with IFN-γ for the indicated times. iNOS expression (Panel A) and phosphorylation of JAK (Panel B) and STAT (Panel C) were assessed by Western blotting. Each blot is representative of at three individual experiments. Each quantified value is expressed as mean ± SEM. (4.86 MB TIF) [file ppat.1001192.s002.tif]
